# Visualizing genomic evolution in *Caenorhabditis* through WormSynteny

**DOI:** 10.1186/s12864-024-10919-6

**Published:** 2024-10-28

**Authors:** Lilly Bouvarel, Dongyao Liu, Chaogu Zheng

**Affiliations:** School of Biological Sciences, The University of Hong Kong, Hong Kong SAR, China

**Keywords:** *Caenorhabditis*, *C. Elegans*, Comparative genomics, Synteny, Nematode, Genomic alignment, Genomic evolution, WormSynteny, Progressive cactus

## Abstract

Understanding the syntenic relationships among genomes is crucial to elucidate the genomic mechanisms that drive the evolution of species. The nematode *Caenorhabditis* is a good model for studying genomic evolution due to the well-established biology of *Caenorhabditis elegans* and the availability of > 50 genomes in the genus. However, effective alignment of more than ten species in *Caenorhabditis* has not been conducted before, and there is currently no tool to visualize the synteny of more than two species. In this study, we used Progressive Cactus, a recently developed multigenome aligner, to align the genomes of eleven *Caenorhabditis* species. Through the progressive alignment, we reconstructed nine ancestral genomes, analyzed the mutational types that cause genomic rearrangement during speciation, and found that insertion and duplication are the major driving forces for genome expansion. Dioecious species appear to expand their genomes more than androdioecious species. We then built an online interactive app called WormSynteny to visualize the syntenic relationship among the eleven species. Users can search the alignment dataset using *C. elegans* query sequences, construct synteny plots at different genomic scales, and use a set of options to control alignment output and plot presentation. We showcased the use of WormSynteny to visualize the syntenic conservation of one-to-one orthologues among species, tandem and dispersed gene duplication in *C. elegans*, and the evolution of exon and intron structures. Importantly, the integration of orthogroup information with synteny linkage in WormSynteny allows the easy visualization of conserved genomic blocks and disruptive rearrangement. In conclusion, WormSynteny provides immediate access to the syntenic relationships among the most widely used *Caenorhabditis* species and can facilitate numerous comparative genomics studies. This pilot study with eleven species also serves as a proof-of-concept to a more comprehensive larger-scale analysis using hundreds of nematode genomes, which is expected to reveal mechanisms that drive genomic evolution in the Nematoda phylum. Finally, the WormSynteny software provides a generalizable solution for visualizing the output of Progressive Cactus with interactive graphics, which would be useful for a broad community of genome researchers.

**Supplementary Information:**

The online version contains supplementary material available at 10.1186/s12864-024-10919-6.

## Background

Synteny refers to the conservation of the order of genes or genomic blocks on the chromosomes from different species. In comparative genomics, visualizing synteny allows the understanding of many evolutionary processes, such as gene duplication, *de novo* gene birth, gene loss, and genomic rearrangement. The nematode *Caenorhabditis elegans* was originally used as a model organism for molecular, cell, and developmental biology [1], but the organism has recently been developed into a genetic model to study both interspecific evolution and intraspecific variation, thanks to the extensive sampling efforts in the past few decades that isolated many species in the *Caenorhabditis* genus and thousands of wild strains of *C. elegans* [2–8]. Comparative genomics studies across species have revealed important mechanisms of evolution, such as the rise of hermaphroditism through the evolution of new F-box proteins [9–11], the expansion of GPCR chemoreceptor families in *C. elegans* and their contraction in the sister species *C. inopinata* [12, 13], and the frequent loss of introns during nematode evolution [14, 15]. However, these previous studies mainly focused on identifying the orthologous genes in particular pathways or gene families, and very few studies analyzed synteny at the whole-genome scale across multiple *Caenorhabditis* species.

For the studies that indeed examined synteny, it was mostly done between two nematode species. For example, the comparison of the genomes of *C. elegans* and *C. briggsae* revealed extensive conservation of chromosomal synteny and large synteny blocks, which was unexpected since the two species diverged 80 ∼ 110 million years ago [16, 17]. The genomes of *C. inopinata* and its sister species *C. elegans* also showed collinearity for ∼ 76% genes, and the synteny breaks mostly due to the expansion of GPCR gene families in *C. elegans* [13]. Synteny analysis between the two species also found *C. inopinata*-specific loss of genes (e.g., *ergo-1*) involved in the siRNA pathway, which functions to suppress transposases; their loss may have enabled the elevated transposase activities and the expansion of transposable elements in *C. inopinata* [13].

Furthermore, the genomes of *C. elegans* and *Pristionchus pacificus*, which belongs to the Diplogastridae and separated from *Caenorhabditis* 100 ∼ 200 million years ago, showed limited synteny due to intrachromosomal rearrangement [18], supporting the idea that the rate of genome rearrangement is much higher in nematodes compared to *Drosophila* [19]. Recent studies suggested that within the *Caenorhabditis* genus, the self-fertilizing species (*C. elegans* and *C. briggsae*) appeared to have gone through more genomic rearrangement than their outcrossing sister species (*C. inopinata* and *C. nigoni*) [20]. Synteny analysis was also carried out between the free-living *C. elegans* and parasitic nematodes, such as *Brugia malayi* (causative agents of lymphatic filariasis in humans) and *Trichinella spiralis* (causing Trichinellosis) [21–23]. In another example, although > 80% of the one-to-one orthologous genes are shared between *Haemonchus contortus* (a common parasitic nematode of ruminants) and *C. elegans* on syntenic chromosomes, there is very little conservation of gene order due to vast rearrangements [24]. Moreover, synteny was used to explore the genomic basis of parasitism in the Strongyloides clade [25–27].

In this study, we analyzed the synteny across eleven *Caenorhabditis* species using a newly developed software called Progressive Cactus, which is a reference-free multi-genome aligner developed to align hundreds of vertebrate genomes with efficiency and accuracy [28, 29]. The algorithm uses a species tree as the guide, reconstructs the genome of the most recent common ancestor of two sister species, and progressively aligns multiple genomes using reconstructed ancestral assemblies. This approach is also robust for contigs-level assemblies, making it possible to perform synteny analysis on genomes that have not been assembled into chromosomes [28]. Previously, we constructed groups of orthologous genes across the eleven *Caenorhabditis* species based on protein sequence information and analyzed the evolutionary history of gene duplication [12]. However, these orthogroups do not contain information of genomic location and syntenic context. Here, we built a web App named WormSynteny to not only visualize the genomic synteny across eleven species but also to integrate the orthogroup assignment with synteny information. The synteny database can be queried using *C. elegans* genes or sequences, and the alignment information can be retrieved in the form of tables and visualized in synteny plots. We showcased the utility of WormSynteny to visualize genomic conservation of single-copy genes (one-to-one orthologues), to identify duplicated genes and differentiate parent and daughter genes, to visualize the evolution of gene structures at the level of introns and exons, and to help improve genome annotations. We envision this tool to be useful in understanding the mechanisms of genomic evolution in nematodes.

## Construction and content

### Generating multi-genome alignment using Progressive Cactus

Employing Progressive Cactus (v2.2.0) [28], we aligned the genomes of eleven *Caenorhabditis* species, including *C. becei* (PRJEB28243), *C. bovis* (PRJEB34497), *C. briggsae* (PRJNA10731), *C. elegans* (PRJNA13758), *C. inopinata* (PRJDB5687), *C. latens* (PRJNA248912), *C. nigoni* (PRJNA384657), *C. panamensis* (PRJEB28259), *C. remanei* (PRJNA57750), *C. tribulationis* (PRJEB12608), and *C. tropicalis* (PRJNA53597), which were selected based on their assembly qualities and were used in our previous studies of gene duplication in *Caenorhabditis* [12]. The eleven genomes and a guide tree from our previous study [12] were used as the input for Progressive Cactus, and the software was ran according to a step-by-step protocol provided at https://github.com/ComparativeGenomicsToolkit/cactus/blob/master/doc/progressive.md. The output file of Progressive Cactus is a HAL file, which is a graph-based representation of the multigenome alignment. The HAL file can be queried using the HAL toolkit (v2.2) [30].

**Fig. 1:**
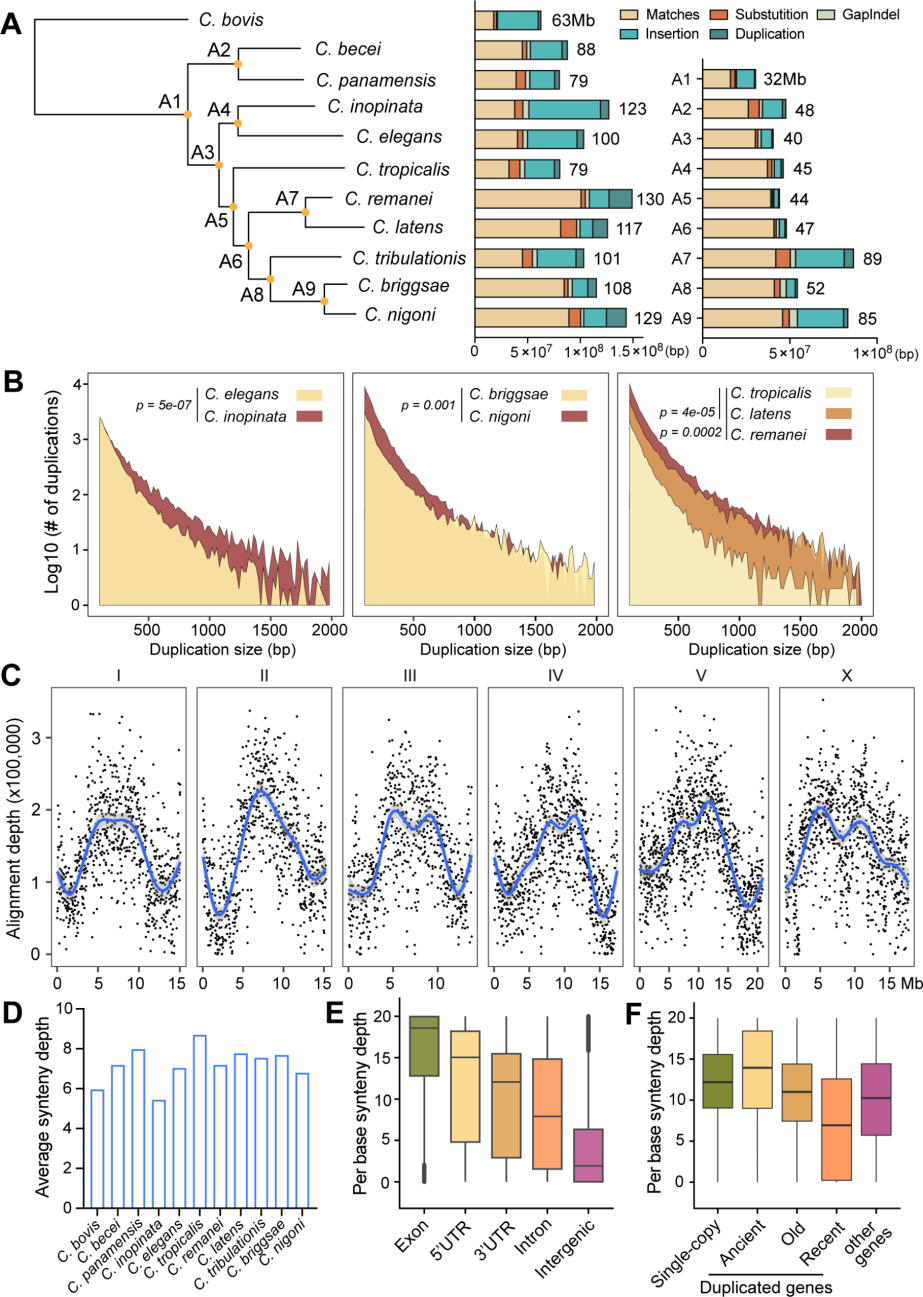
Analysis of progressive cactus output. (**A**) The guide species tree provided to Progressive Cactus for the alignment of eleven *Caenorhabditis* genomes. A1-A9 are the nine ancestral genomes reconstructed by Progressive Cactus. The bar graphs showed the number of bases in the descendant genomes that match the ancestral genomes, and the bases are classified as substitutions, small indels (GapIndels, a combination of GapInsertions and GapDeletions), insertion, and duplication. The numbers for deletions, inversions, and transpositions are small and thus not shown. The left panel shows the statistics for the terminal eleven species, and the right panel for A1-A9. The numbers next to the bar indicate the genome size. (**B**) The comparison of the number of duplication events of various sizes between the self-fertilizing species (*C. elegans*, *C. briggsae*, and *C. tropicalis*) and their outcrossing sister species. Duplications smaller than 50-bp were excluded from the analysis. Paired *t*-test was used to calculate statistical significance between the sister species. (**C**) Alignment depth across the *C. elegans* chromosomes. Each dot represents a non-overlapping 20-kb window. The blue line was generated by fitting the data into a Generalised Additive Model (GAM); the grey areas indicate 95% confidence intervals. (**D**) Average synteny depth per base across the entire genome for the eleven terminal species. (**E**) Box plot for the average synteny depth per base in the exon, 5’UTR, 3’UTR, and intron of genes, as well as intergenic regions. (**F**) Box plot for the synteny depth per base in single-copy genes, duplicate genes that were duplicated at different times, and other genes, which were defined in our previous studies [12]

Progressive Cactus also generated nine ancestral genomes (A1-A9), which can be used to analyze genomic evolution in the genus (Fig. 1A). By comparing the genomes of the eleven terminal species with their most recent ancestral genomes, we found various levels of genomic conservation during terminal speciation. For example, the number of bases that match the ancestral genomes are much higher in *C. remanei* and *C. latens* compared to *C. inopinata* and *C. elegans* (Fig. 1A), which is consistent with an earlier time of divergence for the latter two species.

Among the mutational types (analyzed using the halSummarizeMutations command in the HAL toolkit), large insertions and duplications appeared to be the main force that drove genomic evolution (Fig. 1A and Table S1). The hypothetical ancestral genomes are generally smaller than the descendant genomes, although they may not represent the real ancestors in evolution since large deletions cannot be reconstructed computationally. Insertions appear to cause genome size increase in some cases (e.g., from A4 to *C. inopinata* and *C. elegans*, from A6 to A7, from A8 to A9, and from A8 to *C. tribulationis*), while duplications are more important in driving genome expansion in other cases (e.g., from A7 to *C. remanei* and *C. latens* and from A9 to *C. nigoni*; Fig. 1A). Interestingly, reproductive modes appear to affect the frequency of duplication, since the self-fertilizing species (*C. elegans*, *C. briggsae*, and *C. tropicalis*) had fewer duplicated nucleotides and duplication events than their outcrossing sister species (Fig. 1A and B). For example, *C. elegans* has ∼ 20% fewer duplicated nucleotides than *C. inopinata* (6.3 vs. 7.8 million bp or Mbp) due to fewer large-sized duplications, while *C. briggsae* has > 50% fewer duplicated nucleotides compared to *C. nigoni* (8.2 vs. 18.8 Mbp) because of fewer small-sized duplications (Fig. 1B). Although the self-fertilizing *C. tropicalis* has no clear sister species, comparison with closely related *C. remanei* and *C. latens* revealed significantly fewer duplications of all sizes in the androdioecious species. Our results suggest that the genome size reduction associated with the transition to self-fertilization [31, 32] may be partly due to lower frequency of duplication events.

Next, we calculated the synteny depth in the 11 terminal species and found that the average alignment depth (per base) ranges from 5.4 in *C. inopinata* to 8.7 in *C. tropicalis* with a mean of 7.2, suggesting a moderate level of genomic conservation (Fig. 1D). Using *C. elegans* as an example, we analyzed the synteny depth across chromosomes and found that they share a similar pattern that the chromosomal centers have higher levels of conservation than the arms (Fig. 1C). Interestingly, the chromosomal ends (presumably the telomeres) appeared to be more conserved than adjacent regions on the autosomes. We also surveyed the synteny depth at different sequence types and found that exons are generally more conserved than the untranslated regions (UTRs) and introns, which are more conserved than the intergenic regions (Fig. 1E). However, there are considerable numbers of outliers in exons (reflecting highly diverged or species-specific genes) and intergenic regions (possibly reflecting conserved non-coding or regulatory sequences).

Using the proteomic data, we previously defined three sets of duplicated genes in *C. elegans* based on the time of their duplication [12]. Ancient duplication preceded the separation of the eleven species, old duplication occurred before the separation of the common ancestor of *C. elegans* and *C. inopinata* from the other species, and recent duplication occurred afterwards (Fig. 1F). As controls, we also defined a set of single-copy genes that have only one ortholog in each of the eleven *Caenorhabditis* species [12]. As expected, we found that the recently duplicated genes showed lower synteny depth than the older duplicate genes (Fig. 1F), confirming their more recent origin. Interestingly, ancient duplicate genes have higher synteny depth than single-copy genes, likely because each gene can be aligned to multiple orthologs in other species. The above findings confirmed that the output of Progressive Cactus can provide information about the genomic evolution in *Caenorhabditis* nematodes.

### Constructing an R Shiny App WormSynteny to visualize the genomic alignment

**Fig. 2:**
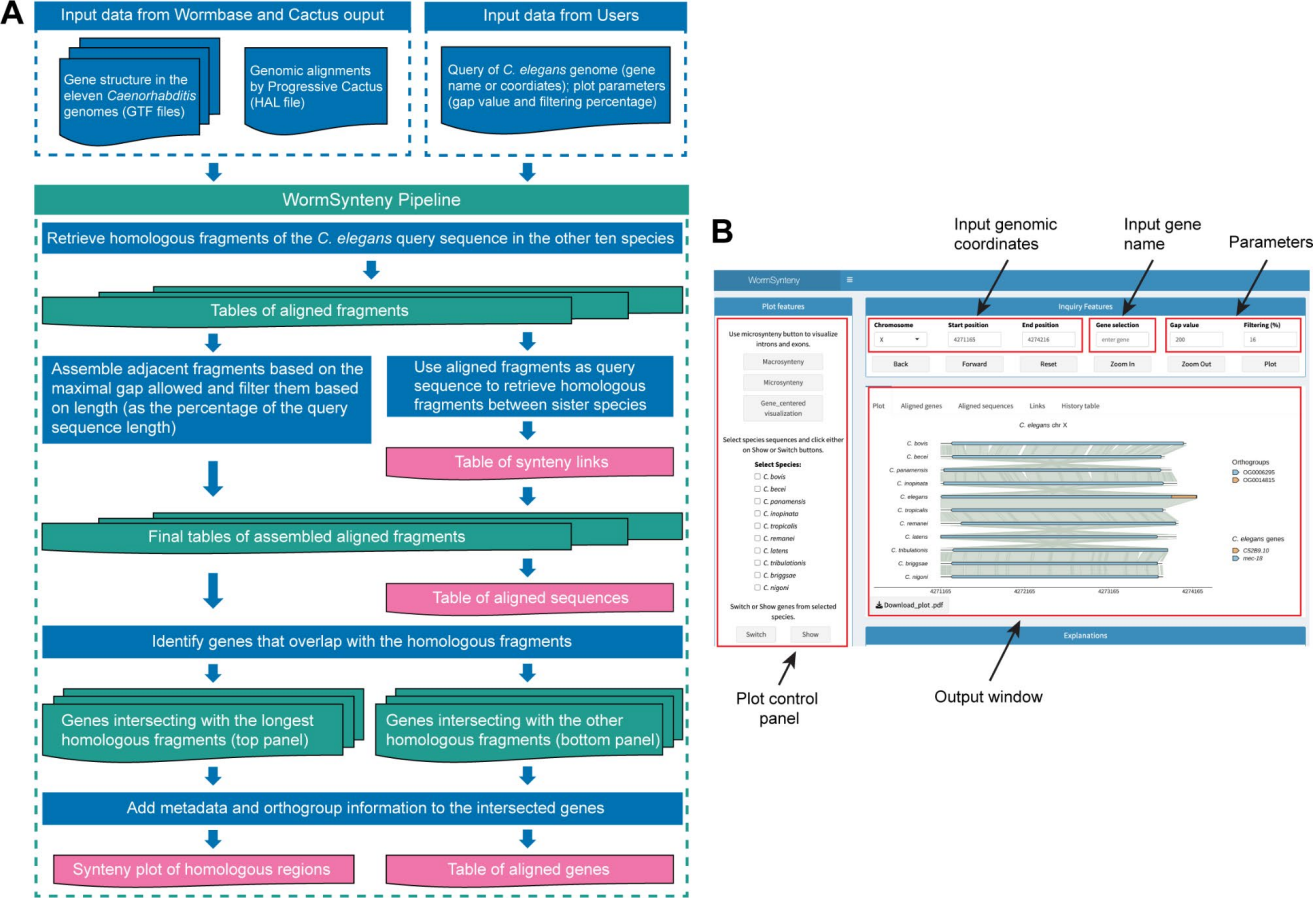
Flowchart and user interface of the WormSynteny App. (**A**) The flowchart of the WormSynteny App. It takes the Progressive Cactus output file and genome annotation files of the eleven species, as well as the query sequence from the user; it then goes through a pipeline to generate four output files (indicated in pink boxes). The pipeline takes *C. elegans* genomic coordinates, searches the HAL file, and exports pairwise alignment information into PSL files using halLiftover. Ten pairwise alignment PSL files are generated for the alignment between *C. elegans* and the other ten *Caenorhabditis* species; these PSL files are then fed into the pipeline to be assembled into long homologous fragments. The coordinates of the fragments aligned to *C. elegans* sequences are extracted to be used as the query sequence to search the HAL file again to find homologous fragments and build the pairwise synteny links between sister species. Thus, ten additional pairwise alignment PSL files are generated to store the alignment information between sister species. (**B**) The layout of the user interface. The top right panel allows the users to input either genomic coordinates or gene name, as well as two parameters (gap value and filtering percentage) to control the output of the pipeline. The bottom right panel contains the outputs, including a gggenomes plot and three tables that contain the alignment information. There is also “History table” tab that allows the users to access output files from previous queries. The top left panel contains some options to customize the visualization of the alignment plot

To visualize synteny at the gene level among the eleven *Caenorhabditis* species, we built an interactive web application named WormSynteny, which is an Ubuntu (22.04.4 LTS)-based app implemented with the R (v4.4.1) Shiny package (v1.8.1.1). The web version of the app can be accessed at https://www.wormsynteny.org, allowing users to query the synteny data. The app takes a *C. elegans* genomic region as the query sequence and extracts the alignment information in the Cactus output using the halLiftover (v2.2) Linux command in the HAL toolkit [30]. It then assembles the aligned segments into larger fragments and filters the fragments by length before plotting them using gggenomes (v1.0.0). The final alignment plot is displayed in the order that follows the species tree, shows annotated genes in blocks, and presents the synteny links between sister species as lines (see the flowchart in Fig. 2A). Since the WormSynteny app is built with a focus on *C. elegans*, the users can only query the database using *C. elegans* gene names or genomic coordinates. The output contains an alignment plot, a list of aligned genes in the eleven nematode species, the coordinates of the aligned sequences, and a table of the synteny links (Fig. 2B).

**Fig. 3:**
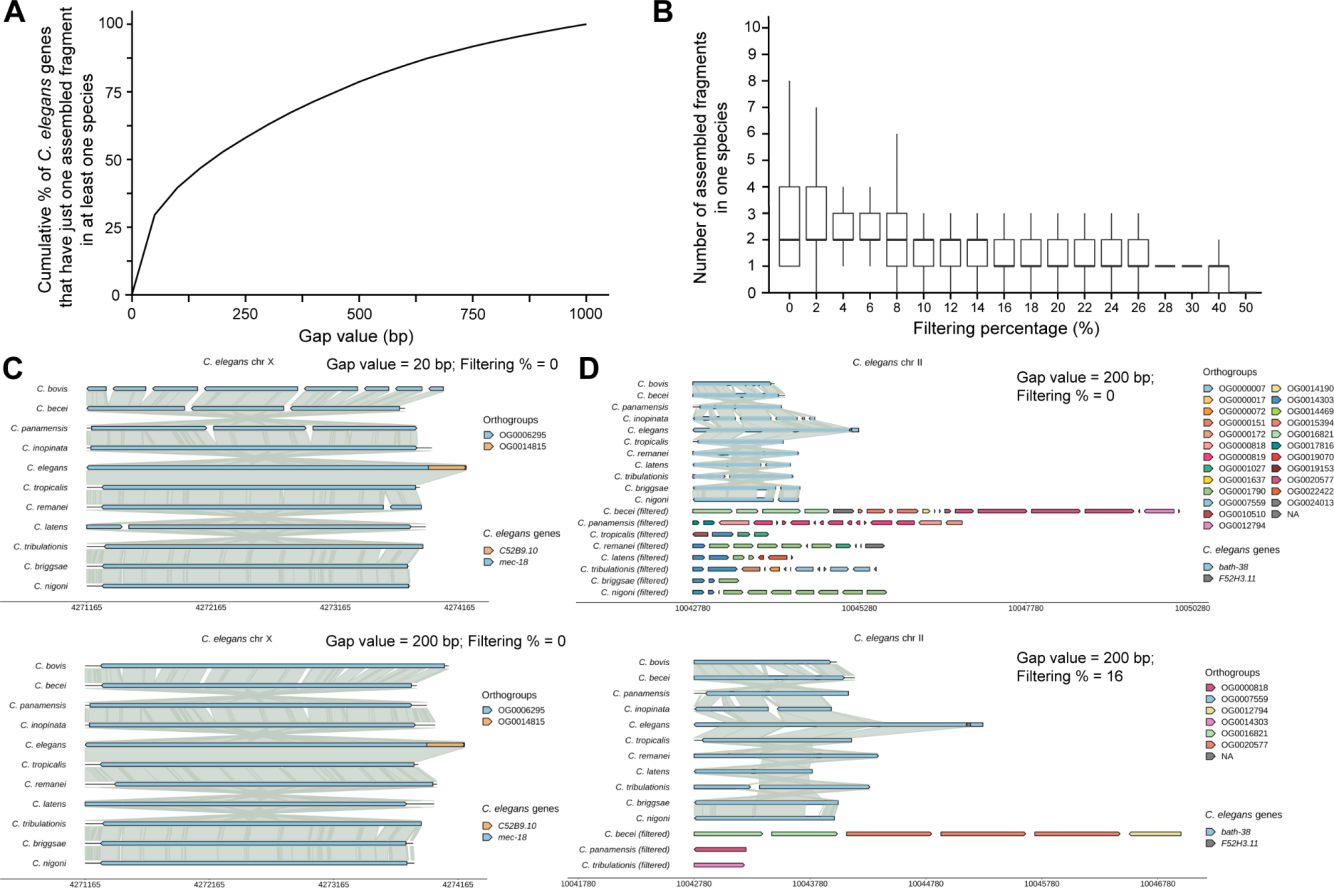
The effects of gap values and filtering percentage on the plot. (**A**) The percentage of *C. elegans* single-copy genes that are aligned to one assembled fragment (i.e., all small fragments are assembled into one) in at least one species at different gap values from 0 to 1000 bps with a 50-bp increment; filtering percentage was set at 0%. (**B**) The average number of assembled aligned fragments per species for the *C. elegans* single-copy genes at different filtering percentages from 0 to 50% with a 2% increment; gap value was set at 200 bp. We calculated the number of assembled fragments left after the filtering in each species (ten numbers for each query) and combined all data for plotting. (**C**) An example of increasing gap value to assemble smaller fragments into one long aligned fragment. “*mec-18*” was inputted in the “Gene selection” field in the WormSynteny app; gap value was set at 20 or 200; filtering percentage was set at 0% for both plots. (**D**) An example of increasing the filtering percentage to remove smaller secondary (or paralogous) alignment. “*bath-38*” was inputted in the “Gene selection” field; filtering percentage was set at 0 or 16; gap value was set at 200 for both plots

In the app, we designed two parameters (i.e., gap value and filtering percentage) that can be adjusted to dynamically control the output of aligned fragments. The gap value is the maximum length of the gap (bp) allowed between two adjacent aligned fragments (output of halLiftover) for their assembly into one larger fragment. Increasing the gap value increases the chance of assembling all the aligned fragments into one aligned region (Fig. 3A). To assess how gap value affects the alignment output, we analyzed the alignment of 4,736 single-copy genes previously defined [12]. We found that with increasing gap values from 0 to 1,000 bp, there was an increasing percentage of *C. elegans* single-copy genes (used as query) that were aligned to a fully assembled fragment in at least one species (Fig. 3A). Since ∼ 50% of the genes were aligned to one fragment when the gap value was set at 200 bp, we used it as the default setting. Nevertheless, we recommend increasing the gap values when the query sequence is longer than 3 kb.

The filtering percentage sets the minimal length (in the percentage of the query length) for the assembled fragment to be included in the final output. Increasing the filtering percentage results in fewer fragments from each species to be presented in the synteny plot (Fig. 3B). When the filtering percentage is set at 16% or above (gap value = 200 bp), there is on average one assembled fragment left for each species. So, we used 16% as the default setting.

The top panel of the synteny plot shows the longest assembled fragment from each species with synteny links between the aligned regions. Smaller fragments that map to the same gene as the longest fragment are also presented in the top panel. Increasing the gap value often allows the assembly of these fragments into one fragment that covers the entire length of the aligned gene (Fig. 3C). Alternatively, users can click the “Full Genes Extension” button on the app to reach the ends of all aligned genes and visualize their full length in the top panel. When the query *C. elegans* gene aligns to more than one gene in another species and the aligned fragment is long enough to pass the filtering threshold, these additional aligned fragments are placed in the bottom panel and labeled with “(filtered)” (Fig. 3D). These secondary aligned genes are potential paralogs of or share some sequence similarity with the main aligned genes. In addition, we built some plot control functions to customize the presentation of the main and extra aligned fragments. The app also allows the users to access all plots in a session through the “History table” tab or to retrieve previous plots using the “Back” and “Forward” buttons. Details can be found in the tutorial on the WormSynteny website www.wormsynteny.org.

## Utility and discussion

### Visualizing syntenic conservation of single-copy genes across species

Using the WormSynteny app, we first assessed the synteny conservation of highly conserved single-copy genes across species. Among the 4,736 single-copy genes, 4,608 genes (97.3%) could be aligned to the orthologs in the same orthogroup (OG) for all ten other species. Since the OGs were generated based on protein sequence similarity (Table S2), our data suggested the conservation of both protein sequences and genomic locations during evolution for vast majority of the single-copy genes. Interestingly, for the 128 cases where the *C. elegans* gene could not be aligned to the ortholog in at least one species, 125 cases were due to the lack of alignment to *C. bovis* ortholog. Given that C. *bovis* is highly diverged from the other *Caenorhabditis* species [33], we reasoned that genomic divergence may have caused the loss of synteny for some conserved genes. Indeed, in 93 of the 125 cases, the *C. elegans* gene had either no alignment to *C. bovis* genome or was aligned to an intergenic region. In the rest 32 cases, the *C. elegans* gene was aligned to a *C. bovis* gene not belonging to the same OG. Manual inspection showed that in some cases (e.g., *set-22*) structural rearrangement of nearby genes disrupted the synteny (Figure S1A), while in other cases (e.g., *rsp-8*) the *C. bovis* gene with syntenic conservation was not assigned to the same OG due to limited protein sequence homology (Figure S1B).

**Fig. 4:**
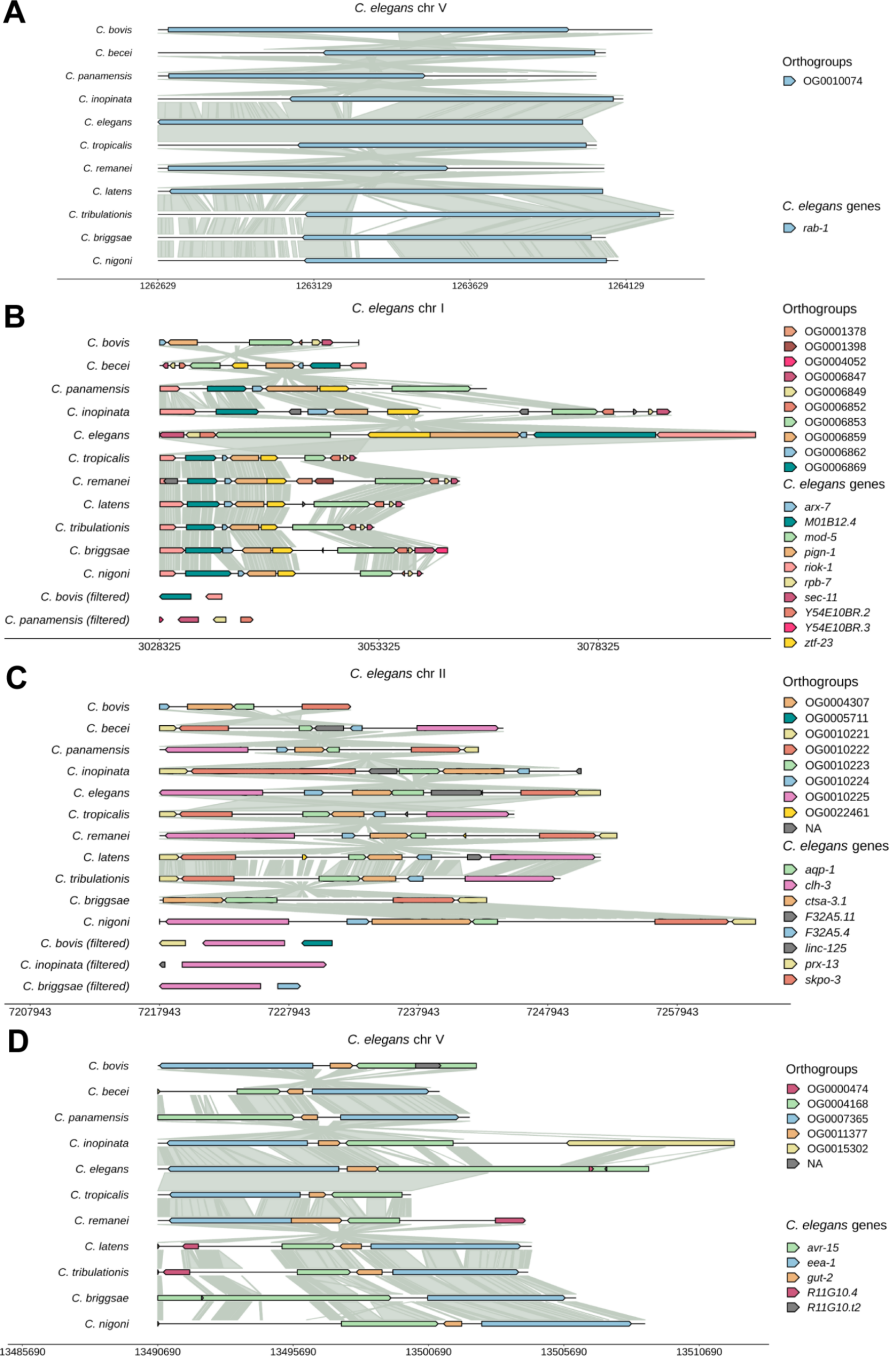
Conservation of synteny for one-to-one orthologs. (**A**) An example of synteny plot for a single-copy gene (defined as genes that have one ortholog in each species; Table S2). “*rab-1*” was inputted into the “Gene selection” field, gap value = 500, filtering % = 0. (**B**) An example of synteny block across the eleven species. Most *C. elegans genes* shown in this block are single-copy genes except for *Y54E10R.3* and *ztf-23*. “I”, “3028325”, and “3096266” were inputted into the “Chromosome”, “Start position”, and “End position” fields, gap value = 10,000, filtering % = 10. (**C**) An example of syntenic block that are partially rearranged in some species. “II”, “7217943”, and “7252015” were inputted into the “Chromosome”, “Start position”, and “End position” fields, gap value = 10,000, filtering % = 10. *aqp-1*, *clh-3*, *F32A5.4*, *prx-13*, and *skpo-3* are single-copy genes. Some orthologs (e.g., *clh-3*) broke off from the syntenic block in *C. bovis*, *C. inopinata*, and *C. briggsae* and are in fragments disconnected from the main assembled homologous fragment. They are labelled as (filtered) in the bottom panel. (**D**) An example of ortholog loss in *C. briggsae*. “V”, “13490690”, and “13508819” were inputted; gap value = 10,000, filtering % = 16. The ortholog of *gut-2* is present in ten species but missing in *C. briggsae*

Apart from the above exceptions involving *C. bovis*, most single-copy orthologous genes showed conserved synteny (Fig. 4A) and are often located in syntenic blocks that contain multiple conserved genes (Fig. 4B-C). Nevertheless, we also observed rearrangement of some genomic blocks, during which one or a few genes may break from the block and relocate to other parts of the genome in certain species (e.g., *clh-3* in Fig. 4C). The syntenic blocks also evolved through the expansion of gene size (e.g., *mod-5*, *pign-1*, and *M01B12.4* in *C. elegans* shown in Fig. 4B), which is often due to intron expansion (see below sections), insertion, deletion, and inversion events.

Moreover, WormSynteny can visualize the loss of conserved one-to-one orthologs in certain species. From the orthogroup data we could identify OGs that have one ortholog in all but one species and use synteny to confirm the species-specific loss of the ortholog in the genome. For example, we found a *C. briggsae*-specific loss of *gut-2*, which codes for a conserved U6 snRNA-associated Sm-like protein (Fig. 4D), confirming the utility of the app to observe gene loss.

### Visualizing gene duplications

**Fig. 5:**
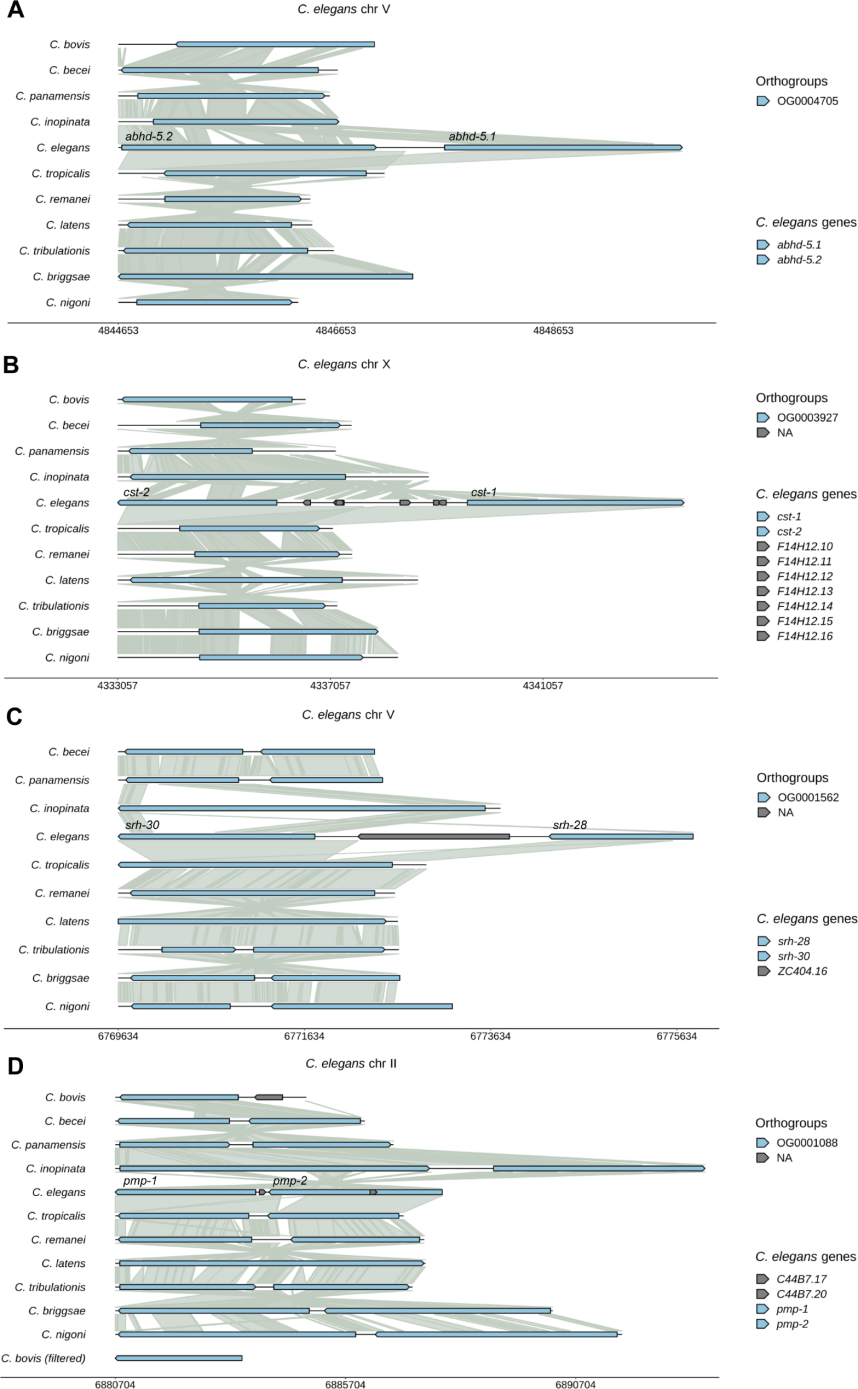
Visualizing tandem duplication using WormSynteny. (**A**) *C. elegans*-specific tandem duplication created *abhd-5.1* and *abhd-5.2* genes. “V: 4844653 . 4849838” was inputted as the genomic coordinates. All other species have only one ortholog in this orthogroup (OG0004705), and Progressive Cactus did not pick up secondary aligned regions. (**B**) *C. elegans*-specific duplication generated *cst-1* and *cst-2* genes which are separated by small noncoding RNAs (F14H12.10 ∼ F14H12.16). “X: 4333057 . 4343700” was used as the input. (**C**) An example where the duplication that generates *srh-28* and *srh-30* occurs in previously defined “old” time (after the separation of *C. bovis* and A1 but before the separation of A4 and A5). Synteny matches the orthogroup assignment and shows that the tandem duplication occurs in multiple species except for *C. bovis* (no alignment in *C. bovis* was found), but one paralog is lost, or the two paralogs are fused in *C. inopinata*, *C. tropicalis*, *C. remanei*, and *C. latens*. “V: 6769634 . 6775806” was used as the input. (**D**) An example of “ancient” duplication that occurred before the separation of *C. bovis* and other species. “II: 6880704 . 6887800” was used as the input. Two paralogs can be found in all species except for *C. latens*, which may have lost one paralog due to gene fusion. The two paralogs in *C. bovis* are not adjacent to each other; they are > 700 kb away from each other. In (A-C), gap value = 500, filtering % = 16. In (D), gap value = 2000, filtering % = 16. In (A-D), the “Full gene extension” function was used to reach the ends of the orthologs

In addition to the single-copy genes, WormSynteny can visualize duplication events. For example, we identified 766 orthogroups in which *C. elegans* has two paralogs, among which 76 OGs have only one ortholog in the other ten species, indicating *C. elegans*-specific duplication events (Table S3 and S4). These *C. elegans* paralogous pairs are usually adjacent genes (e.g., *abhd-5.1* and *abhd-5.2* in Fig. 5A) or sometimes separated by non-coding genes (e.g., *cst-1* and *cst-2* in Fig. 5B), suggesting that they are derived from tandem duplications. We also examined cases where the duplication occurred at an earlier time (e.g., ancient or old duplication before the separation of ancestral A4 and A5), and synteny revealed the presence of duplicate genes in several species, while the others have only one copy likely due to gene loss or gene fusion (e.g., *srh-28* and *srh-30* in Fig. 5C and *pmp-1* and *pmp-2* in Fig. 5D). The two scenarios can be differentiated by observing the synteny links (i.e., one ortholog showing synteny to only one paralog in sister species may suggest gene loss or lack of duplication, while synteny to two paralogs may indicate gene fusion).

Besides tandem duplication, synteny can also help visualize dispersed duplications. For example, *akt-1* and *akt-2* in *C. elegans* are located on chromosome V and X, respectively, and both genes are aligned to the same regions on chromosome V in the other ten species, suggesting that *akt-2* was likely derived from a duplication of *akt-1* and then inserted into chromosome X (Figure S2). This dispersed duplication also occurred in the sister species *C. inopinata*, but its two paralogs are still located on the same chromosome, although ∼ 26 kb apart from each other.

Moreover, local synteny and the alignment (PSL) score provided the possibility to identify potential parental and daughter genes (Table S4). The PSL score is calculated based on the formula (PSL = matches + repMatches – mismatches – qNumInsert – tNumInsert) and is then normalized to the length of the paralog. For example, the cystathionine beta-synthase gene had a *C. elegans*-specific duplication, which gave rise to two paralogs *cbs-1* and *cbs-2*; both are aligned to the same orthologs in the other ten species (Figure S3A-D). The alignment score for *cbs-1* is higher than *cbs-2* (when aligned to *C. inopinata* ortholog, PSL score is 1,438 for *cbs-1* and 187 for *cbs-2*; the average PSL scores in the alignment with the ten other species is 1,362 for *cbs-1* and 149 for *cbs-2*). The local synteny showed good conservation of gene order for *cbs-1* and the neighboring genes on chr X, whereas *cbs-2* appears to be derived from an insertion on chr II that occurred specifically in *C. elegans* (Figure S3E-F). These findings led to the hypothesis that *cbs-1* is the ancestral gene, while *cbs-2* is the daughter gene generated through duplication.

### Visualizing exon and intron evolution

**Fig. 6:**
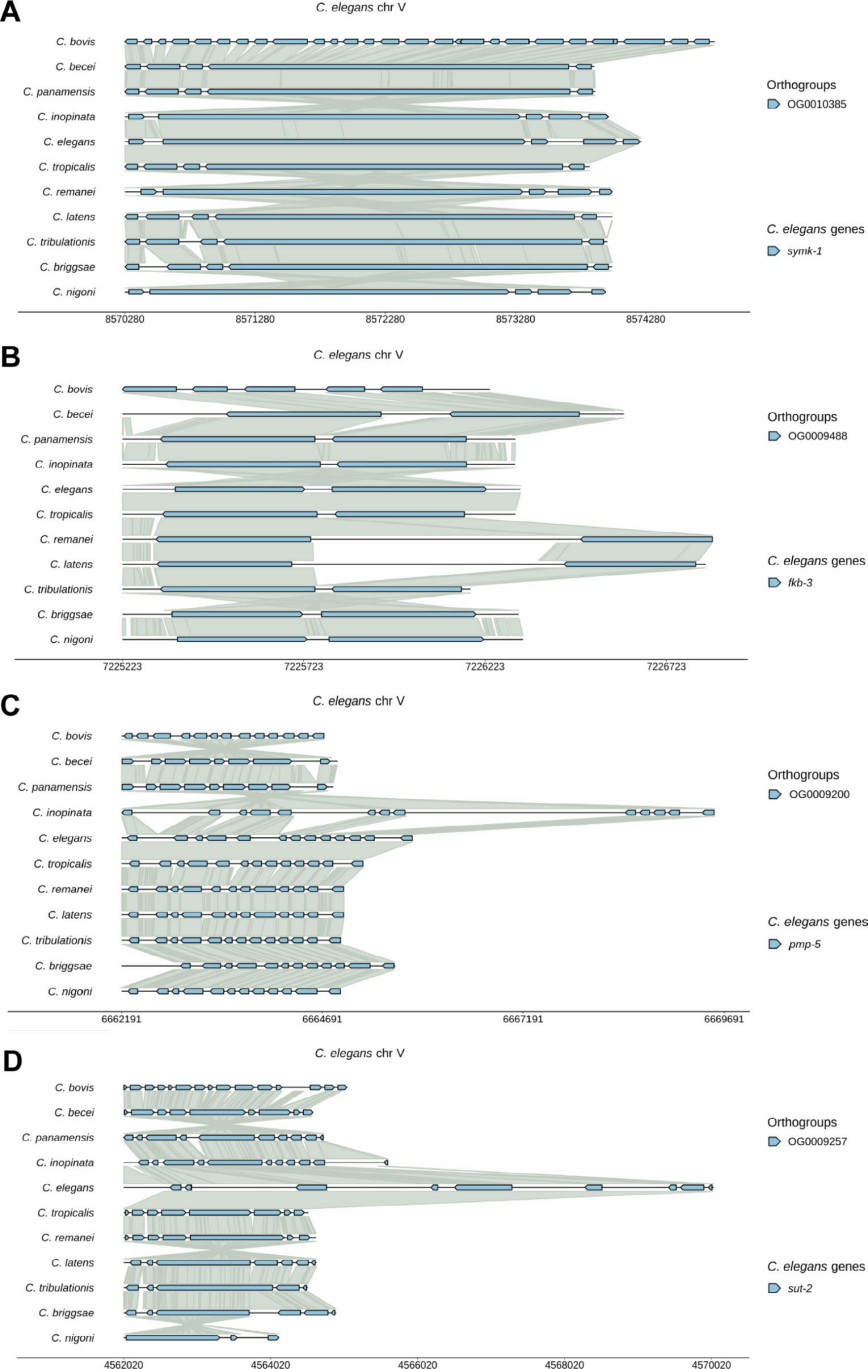
Visualizing synteny at the exon and intron levels using the microsynteny function. (**A**) An example of increased exon numbers in *C. bovis* compared to other species. “*symk-1*” was inputted into the “Gene selection field”. Gap value = 200, filtering % = 16. After the plot was generated, “Full gene extension” and “microsynteny” functions in the plot control panel were used to generate the plots in this Figure. (**B**) An example of increased exon numbers in *C. bovis* and elongated introns in *C. remanei* and *C. latens* among *fkb-3* orthologs. (**C**) An example of intron elongation in *C. inopinata* for *pmp-5* ortholog. (**D**) An example of *C. elegans*-specific intron elongation in *sut-2*

At the individual gene level, WormSynteny can visualize the evolution of exon and intron structures (defined as microsynteny in this study). By examining the 4,736 single-copy genes, we found 1,581 (33%) cases where the *C. bovis* gene had more exons than its orthologs in other *Caenorhabditis* species. In an extreme case, *C. bovis symk-1* has 29 exons, whereas the orthologs in other ten species have only 5 exons (Fig. 6A), indicating possible exon fusion events in the ancestor of the other species after its separation from *C. bovis*. Nevertheless, the sizes of the orthologous proteins are similar across species (e.g., 1,403 amino acids in *C. bovis* and 1,143 amino acids in *C. elegans*). The exon fragmentation of conserved genes in *C. bovis* is somewhat unexpected, since *C. bovis* has the smallest genome among the published *Caenorhabditis* genomes [33].

Another common theme of gene structure evolution was species-specific intron expansion. We observed 524 examples of single-copy genes where either the *C. remanei* or *C. latens* gene has longer introns than other orthologs, and 199 cases where orthologs from the two species had the longest and second longest introns. For instance, the only intron in *fkb-3* (Fig. 6B) and the two introns in *ima-1* (Figure S4A) are significantly expanded in *C. remanei* and *C. latens*, leading to increased gene length in both species. We also found cases where introns are elongated specifically in other species such as *C. inopinata* (e.g., *pmp-5* in Fig. 6C) and *C. elegans* (e.g., *sut-2* in Fig. 6D). In these two examples, only selected introns are elongated in one species. On the other hand, the lengths of most introns in *mod-5* vary significantly across the eleven *Caenorhabditis* species (Figure S4B), suggesting more dynamic intron expansion and contraction. In addition to increasing genome size by expanded introns, previous studies hypothesized that introns could provide a source for the evolution of new genes [34]. Supporting this hypothesis, we found a case where the expanded intron of *C. remanei* and *C. latens twk-11* orthologs evolved a new gene that is unique to the two species and does not have paralogs in the other species (Figure S4C; OG0022526 only contains the *C. remanei* and *C. latens* orthologs shown). Intron expansion can also be associated with local rearrangement, as we found an example where the elongated intron of *C. briggsae glr-5* housed two genes that were adjacent to *glr-5* in *C. elegans* (Figure S4D).

Overall, detecting the change of exon and intron structures at the level of microsynteny among species can help understand the evolution of gene function and expression regulation.

### Improving genome annotation by visualizing synteny

**Fig. 7:**
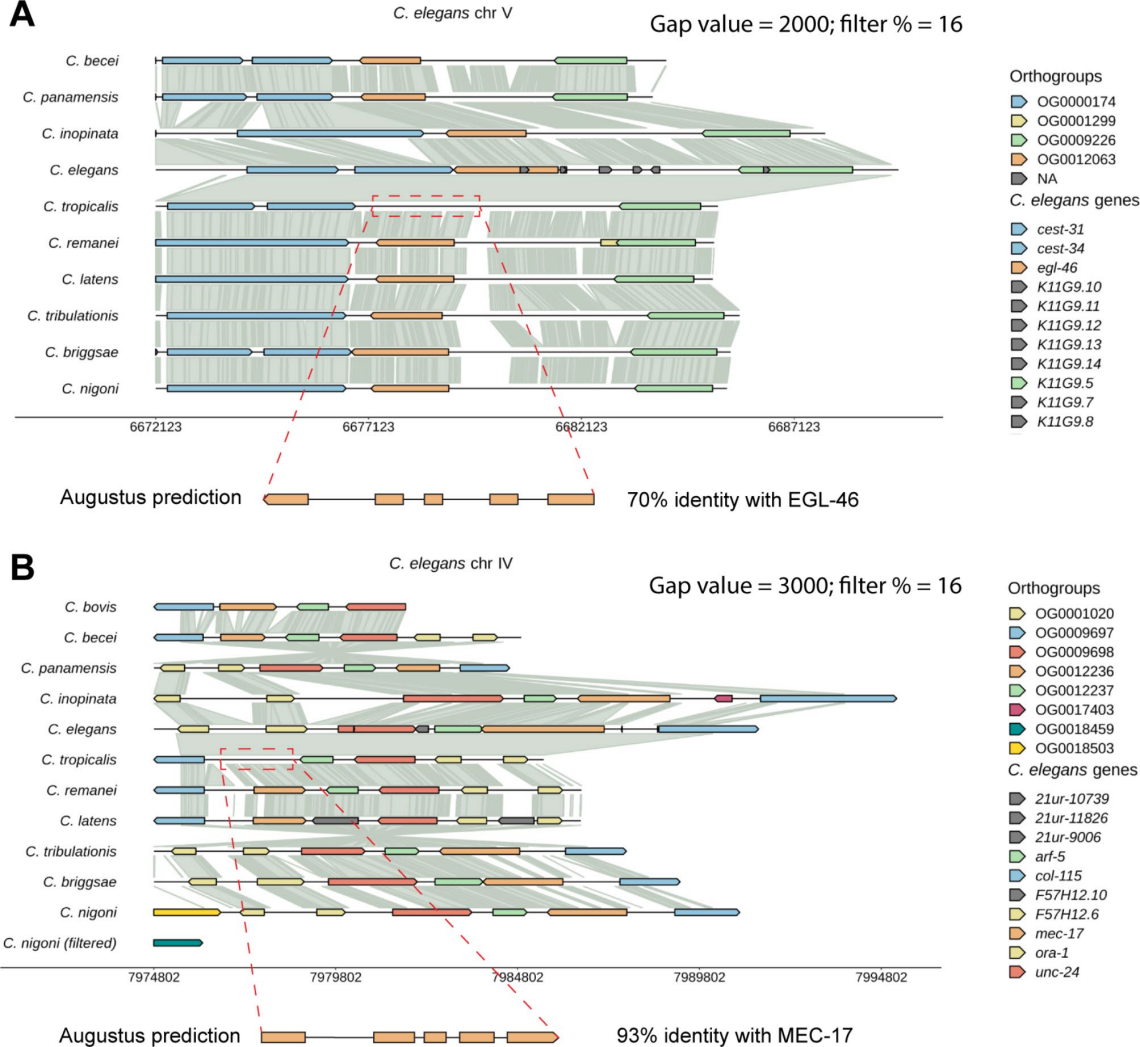
Synteny reveals potential genes missed in the genome annotation. (**A**) “V: 6,672,123 . 6,688,492” was inputted as query *C. elegans* genomic coordinates. The ortholog of *egl-46* is absent in *C. tropicalis*, although conserved synteny can be observed in the intergenic region. The sequence of *C. tropicalis* Scaffold601: 77,339 . 80,338 (red dashed box region) was subjected to gene prediction by Augustus, which annotated a gene that contains five exons (like *C. elegans egl-46*) and codes for a protein that has 70% identity with *C. elegans* EGL-46. (**B**) “IV: 7,974,802 . 7,991,428” was inputted as query coordinates. The ortholog of *mec-17* is absent in *C. tropicalis*, and the intergenic region Scaffold629: 19,019,142 . 19,021,447 (red dashed box) was subjected to gene prediction by Augustus, which annotated a gene that codes for a protein that has 93% identity with *C. elegans* MEC-17

Lastly, visualizing synteny can also help improve the annotation of genomes. Using WormSynteny, we have discovered several cases where orthologs were absent in one species in genomic regions that had very good syntenic conservation across species. For example, the ortholog of the transcription factor *egl-46* is missing in the genome of *C. tropicalis*, despite the conserved gene order and good syntenic links in the region. We suspected that the ortholog is absent due to errors in annotation. So, we reannotated this region of the *C. tropicalis* genome using Augustus (https://bioinf.uni-greifswald.de/augustus/submission.php) and identified a gene that has a similar five exon structure as *C. elegans egl-46* and codes a protein with 70% identity to EGL-46 (Fig. 7A). Similarly, we recovered the missing ortholog of the alpha-tubulin acetyltransferase MEC-17 in the *C. tropicalis* genome by visualizing the synteny (Fig. 7B). Thus, we propose to systematically improve the annotation of *Caenorhabditis* genomes by analyzing the conservation of genomic blocks. WormSynteny can be instrumental in this reannotation process.

## Discussion and future development

The advance in various high-throughput DNA sequencing technologies have launched genomic research into the thousand-genome era. For example, Vertebrate Genomes Project aims to sequence ∼ 70,000 extant vertebrate species [35], while the Earth BioGenome Project has the ambition to sequence the genomes of all eukaryotes over a period of 10 years [36]. The nematodes present a unique opportunity to understand genomic evolution in eukaryotes since they have compact genomes, short life cycle, transparent bodies for anatomic studies, and high amenability to genetic manipulation. So far, hundreds of nematode genomes have been sequenced [37, 38], making it possible for large-scale comparative studies. However, a crucial hurdle for such comprehensive analysis is the ability to align hundreds of genomes and visualize the syntenic relationships among them to infer genomic evolution. In this study, we provide a proof-of-concept to use the newly developed multi-genome aligner, Progressive Cactus, to align more than ten nematode genomes, retrieve the ancestral genomes, and obtain insights on genomic rearrangement during speciation.

The online app WormSynteny greatly facilitates the visualization of the syntenic relationship. Although Wormbase (www.wormbase.org) offers the JBrowse 2 Synteny tool (currently in beta) to visualize the synteny between two selected assemblies [39], WormSynteny can simultaneously visualize the synteny among eleven species while offering a set of options to query the alignment information and control the presentation of the synteny plots. These customization tools could become handy when studying synteny at different scales. For example, when analyzing gene gain or loss across a large genomic block, one can use the macrosynteny option to present genes in one block, whereas the microsynteny option is useful to visualize the evolution of gene structure at the exon and intron level. Moreover, by adjusting the filtering percentages, one can obtain the information of both primary and secondary alignment for the query sequence and switch between them when plotting the synteny; the second aligned fragment often contains a paralog of the first aligned gene, which could indicate a potential gene duplication event. These features are not available with the JBrowse 2 Synteny tool.

More importantly, WormSynteny integrates genomic and proteomic information to allow easy identification of syntenic conservation of homologous genes. By constructing orthogroups using only protein sequences [12] and synteny using only genomic sequences (this study), the app can assess whether orthologous genes (which are conveniently labeled in the same color) are located in conserved syntenic blocks across species. One application of this feature is to differentiate tandem duplication from dispersed duplication and to discern ancestral paralog from the newly evolved paralog. We expect WormSynteny to be useful in visualizing the genomic mechanisms underlying the gain and loss of paralogs thanks to the incorporation of independent orthogroup information.

One caveat of the WormSynteny app is that users can only use *C. elegans* genes or genomic coordinates to query the alignment data. We reason that most users will be interested in the synteny information of genes orthologous to a *C. elegans* gene because *C. elegans* is the most experimentally tractable organism among the eleven species and there is a rich literature on the functions of *C. elegans* genes. The *C. elegans* genome is also the best annotated among the eleven. Thus, we prioritized on using *C. elegans* genomic sequences as the query sequence. In the future updates, we can include the option of using the genomic coordinates of other species as the query sequence, especially after the genomic annotation improves in these species.

Overall, through this pilot study of eleven species, we hope to set up a pipeline that can be used to align hundreds of nematode genomes and analyze genomic evolution at a larger scale in the Nematoda phylum. We envision that the tools generated in this study can be immediately useful for the community to analyze syntenic conservation and genomic rearrangement among the most widely used *Caenorhabditis* species. At the same time, our work lays the foundation for future studies on the evolution of nematodes involving potentially hundreds if not thousands of genomes. Lastly, the WormSynteny app provides the first example of visualizing the output of Progressive Cactus with an interactive user interface and has the potential to be further developed into a general graphic solution for presenting the alignment information downstream of Progressive Cactus.

## Conclusions

In this study, we created the genomic alignment across eleven *Caenorhabditis* species and built an online interactive R Shiny app, WormSynteny, to enable users to query the alignment information. Analysis of mutational types found that insertion and duplication drive differential expansion of genomes in self-fertilizing and outcrossing species. Genome-wide characterization of synteny depth revealed that the chromosomal centers are more conserved than the arms, coding regions are more conserved than intergenic regions, and one-to-one orthologs and old duplicate genes are more conserved than young duplicate genes. Using WormSynteny, we were able to visualize the syntenic conservation of single-copy genes, tandem and dispersed gene duplication, and species-specific exon fragmentation and intron elongation. WormSynteny can also help improve genomic annotation and is a useful tool for comparative genomics and the study of nematode evolution.

## Electronic supplementary material

Below is the link to the electronic supplementary material.

Supplementary Material 1

Supplementary Material 2

Supplementary Material 3

Supplementary Material 4

Supplementary Material 5

## Data Availability

Data is provided within the manuscript or supplementary information files. The online version of the WormSynteny app can be accessed at https://www.wormsynteny.org. The website also contains a detailed tutorial on the WormSynteny app and orthogroup information. The source code for the WormSynteny app (version 1.0), which is an R package, can be found at https://github.com/Zhenglabhku/WormSynteny/tree/main/WormSynteny_Package. The stable release of version 1.0 is in the commit be83ab8. We recommend heavy users install an offline version of the app, which may run faster than the web-based tool. The codes used to perform the analysis presented in this paper can be found at https://github.com/Zhenglabhku/WormSynteny/tree/main/Paper. All other relevant information is available upon reasonable request from C.Z. (cgzheng@hku.hk).
