# Supplementary material for: Visualizing genomic evolution in Caenorhabditis through WormSynteny
Source: BMC Genomics. 2024 Oct 28;25:1009. doi: 10.1186/s12864-024-10919-6 (PMC11520455; doi:10.1186/s12864-024-10919-6)
Supplement: Supplementary file 5 — Supplementary Material 5 [file 12864_2024_10919_MOESM5_ESM.docx]

**Supplementary information**


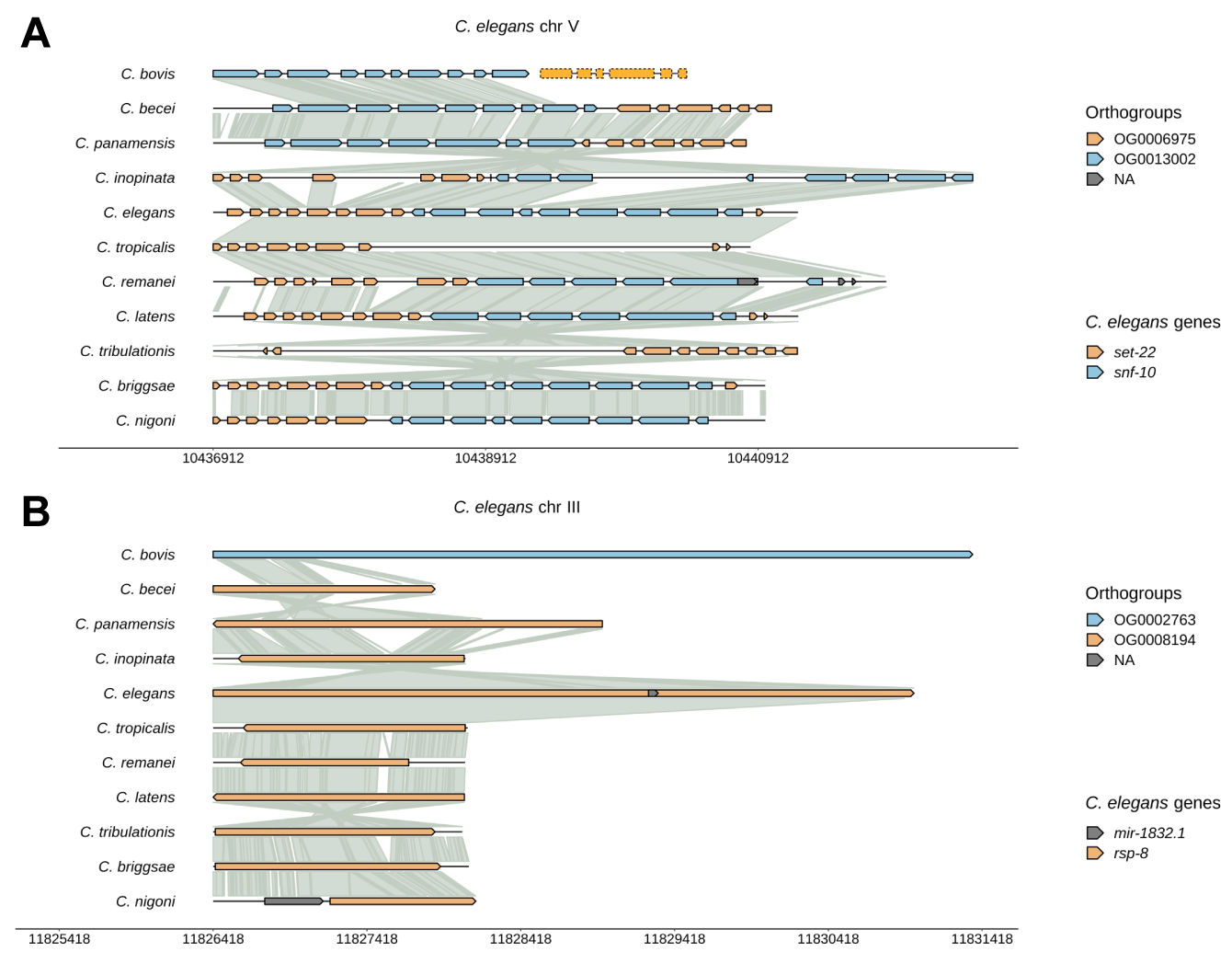


**Figure S1. Examples of missing the alignment to *C. bovis* orthologs for single-copy genes.** (A) “*set-22*” was inputted in the “Gene Selection” field, gap value = 200, filtering % = 16. “Full Genes Extension” and “microsynteny” buttons were pressed on the app to generate the plot. Another gene *snf-10* is nested in the last intron of *C. elegans* *set-22*, which led to the alignment to both genes in other species. In *C. bovis*, the two genes are no longer nested, leading to the failure of discovering the *set-22* ortholog by WormSynteny. The yellow gene structure with dashed outline indicates that the *set-22* ortholog is still adjacent to the *snf-10* ortholog in *C. bovis*. (B) “*rsp-8*” was inputted into the “Gene Selection” field, gap value = 1000, filtering % = 10. “Full Genes Extension” function was used; additional alignments were not shown by pressing the “Hide” button on the App. The *C. bovis* ortholog shown in the plot is CBOVI.g5204, which is different from the ortholog CBOVI.g11711 assigned in proteome-based orthogroups. *C. elegans* RSP-8 protein has 309 a.a. and shares 13.4% identity and 14.8% similarity with CBOVI.g5204 (1127 a.a.) and 12.3% identity and 17.8% similarity with CBOVI.g11711 (88 a.a.). The low sequence similarity may lead to the misassignment of orthologs in the orthogroups.

**
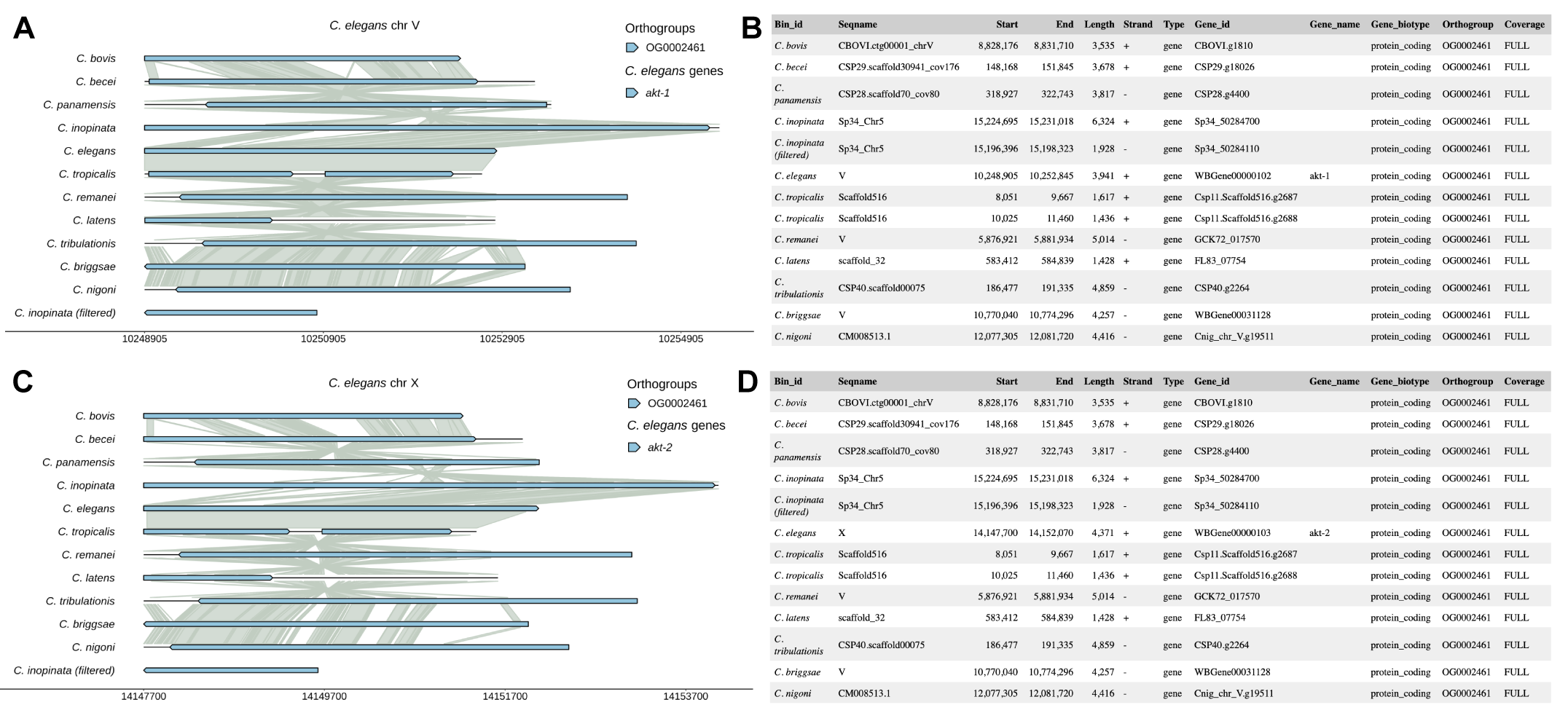
**

**Figure S2. Dispersed duplication of *akt-1* and *akt-2*.** (A) “*akt-1*” was inputted in the “Gene Selection” field, gap value = 1000, filtering % = 16, and “Full Genes Extension” were used. (B) The output table of “aligned genes”. *akt-1* is located on chromosome V. Note that *akt-1* is aligned to two genes in *C. inopinata* that are both on Chr5 but 26 kb apart. (C) “*akt-2*” was inputted in the “Gene Selection” field, gap value = 1000, filtering % = 16, and “Full Genes Extension” were used. *akt-2* is located on chromosome X and is aligned to the same Chr V-located orthologs in the other ten species as *akt-1*. (D) The output table of *akt-2* “aligned genes”, which is the same as that of *akt-1*.

**
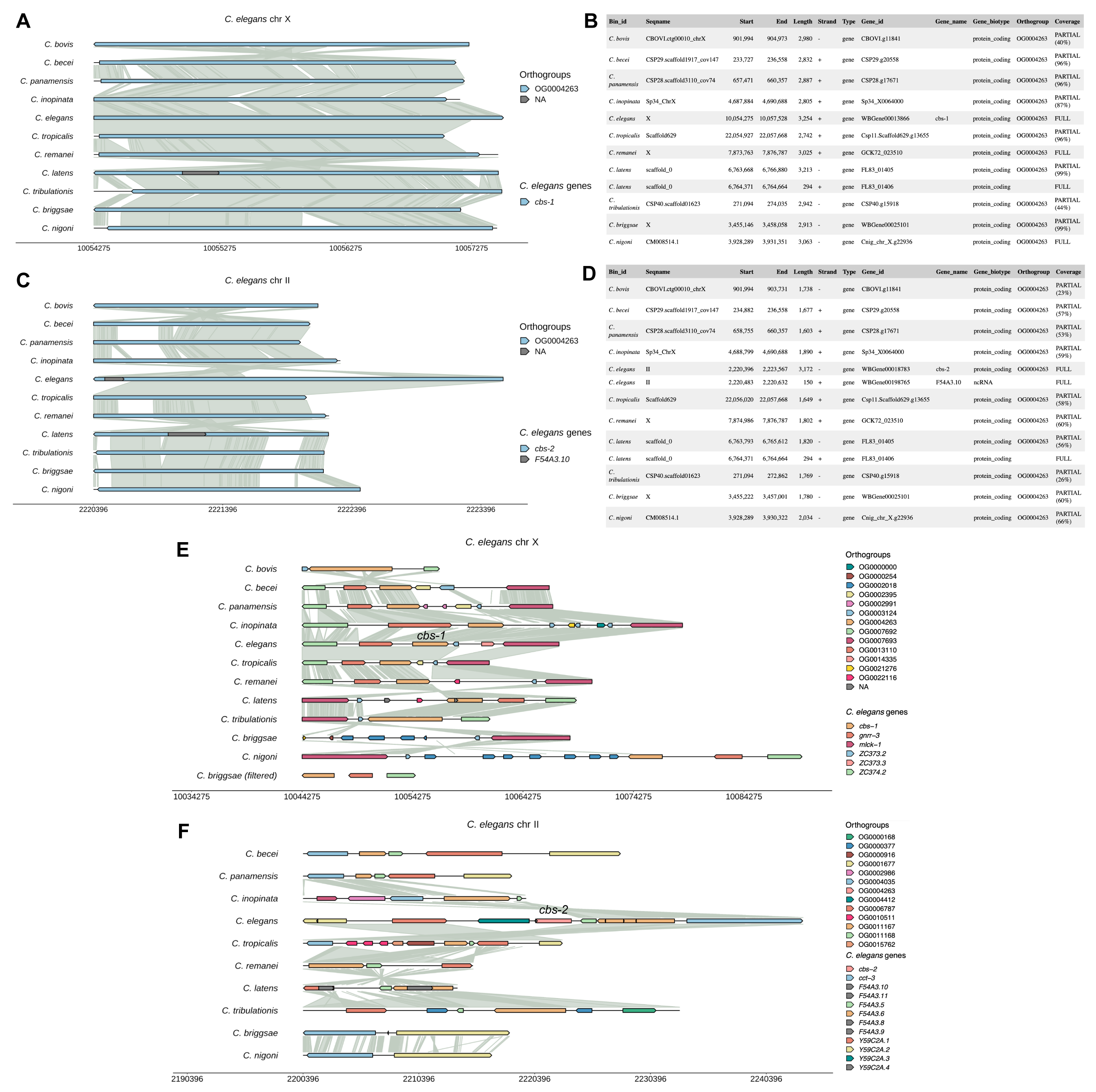
**

**Figure S3. Discerning parent and daughter genes using WormSynteny.** (A) “*cbs-1*” was inputted in the “Gene Selection” field, gap value = 500, filtering % = 16, and “Hide” function was used. (B) Output table of “aligned genes” for *cbs-1*. *cbs-1* and the aligned genes are all located on Chr X. (C) “*cbs-2*” was inputted in the “Gene Selection” field, gap value = 500, filtering % = 16, and “Hide” function was used. (D) Output table of “aligned genes” for *cbs-2*, which is the same as the table for *cbs-1* in (B). *cbs-2* is located on Chr II but the aligned genes are on Chr X. (E) “X: 10,044,275 .. 10,069,792” was inputted as genomic coordinates. Gap value = 10,000, filtering % = 16. (F) “II: 2,200,396 .. 2,243,539” was inputted as genomic coordinates. Gap value = 10,000, filtering % = 16. “Hide” function was used to remove the smaller aligned fragments.

**
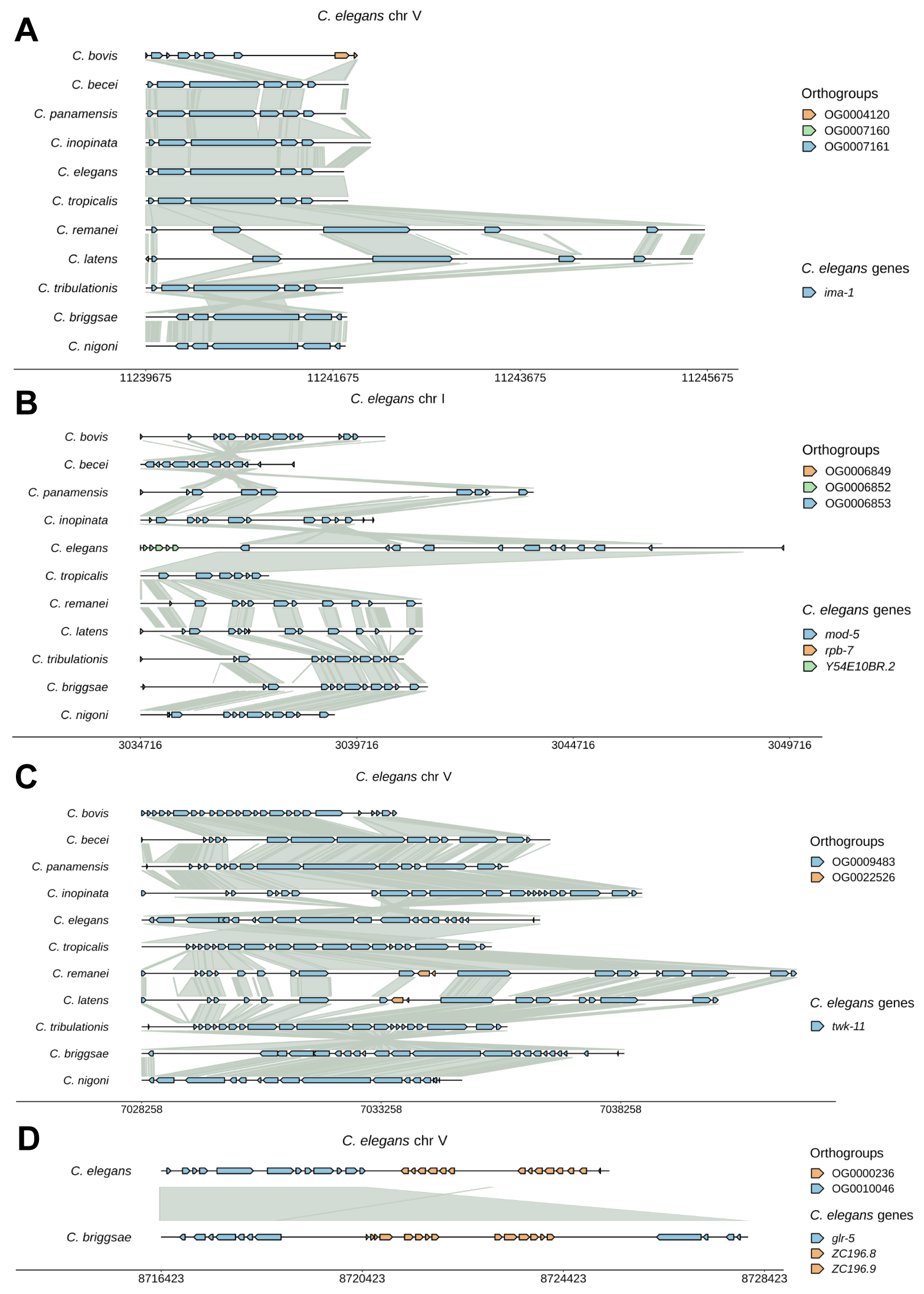
**

**Figure S4. Examples of evolution of intron and exon structures.** (A) An example of intron elongation in *C. remanei* and *C. latens* orthologs of *ima-1*. “*ima-1*” was inputted into the “Gene Selection” field. Gap value = 2000, filtering % = 16, and “microsynteny” function was used. (B) An example of elongation of specific introns in *C. elegans* *mod-5* among the orthologs. “*mod-5*” was inputted into the “Gene Selection” field, gap value = 2000, filtering % = 16, “Full Genes Extension” and “microsynteny” functions were used. (C) An example that new genes have evolved in the expanded introns of *C. remanei* and *C. latens* orthologs of *twk-11*. “*twk-11*” was inputted into the “Gene Selection” field, gap value = 2000, filtering % = 16; “Full Genes Extension” and “microsynteny” functions were used. Orthogroup OG0022526 only contains one *C. remanei* gene (GCK72_017304) and one *C. latens* gene (FL83_09705) shown in the graph. (D) An example that expanded intron of *C. briggsae glr-5* harbors nearby genes. “*glr-5*” was inputted into the “Gene Selection” field, gap value = 1000, filtering % = 16, “Full Genes Extension”, “Hide”, and “microsynteny” buttons were pressed to create the plot. Only “*C. briggsae*” was selected from the sidebar, and “Show” function was used to generate the synteny plot just between *C. elegans* and *C. briggsae*.
